# Multiple Looks of Auditory Empty Durations Both Improve and Impair Temporal Sensitivity

**DOI:** 10.3389/fnhum.2018.00031

**Published:** 2018-02-02

**Authors:** Tsuyoshi Kuroda, Daiki Yoshioka, Tomoya Ueda, Makoto Miyazaki

**Affiliations:** ^1^Faculty of Informatics, Shizuoka University, Hamamatsu, Japan; ^2^Yamaha Motor Co., Ltd, Fukuroi, Japan; ^3^Department of Informatics, Graduate School of Integrated Science and Technology, Shizuoka University, Hamamatsu, Japan

**Keywords:** temporal sensitivity, multiple-look effect, rhythm, prediction, regularity

## Abstract

Discrimination of two neighboring empty durations that are marked by three successive sounds is improved when the presentation of the first (standard, S) duration is repeated before that of the second (comparison, C), as SSSSC. This improvement in sensitivity, called the multiple-look effect, has been explained by a statistical model regarding variability. This model assumes that the perceived duration of the standard is averaged across observations (within a trial within an individual). The increasing of the number of observations thus reduces the standard error of the mean perceived duration. Alternatively, the multiple-look effect is attributed to the listener’s prediction based on regular rhythm. Listeners perceive regular rhythm during the repetition of the standard, predict the timing of subsequent sounds, and detect a sound that is displaced from the predicted timing. These models were tested in the present experiment in which the main factor was a temporal separation between the standard and the comparison; i.e., these durations were adjacent to each other as SSSSC or separated by a temporal blank as SSSS_C. The results differed between stimulus structures. First, the multiple-look effect was replicated in the SSSSC condition (yielding a higher performance than SC), but disappeared in SSSS_C (having no difference with S_C). Second, no multiple-look effect occurred in CSSSS (no difference with CS), and moreover, an impairment effect was observed in C_SSSS (a lower performance than C_S). Finally, discrimination was improved in SSSS_CCCC compared with SSSSCCCC, the effect being kept even when sounds were aligned at irregular intervals. These findings are not consistent with those expected from the statistical model because the temporal separation should have produced no effects if the number of standards had been a sole parameter determining the multiple-look effect. The prediction-based model can explain the first finding; inserting a blank between the standard and the comparison violates the listener’s prediction based on regular rhythm, thus reducing the multiple-look effect. However, it did not expect the other findings and required revisions. Notably, the second finding indicates that the formation of regular rhythm can impair temporal discrimination. In other words, an *inversed* multiple-look effect occurs.

## Introduction

People utilize rhythm for predicting subsequent events. Rhythm is formed by the repetition of identical temporal structures, and thus, the perception of a certain type of rhythm informs that a temporal structure appears again and again in the future. In music, a sudden change of rhythm violates listeners’ prediction, resulting in emotion such as surprise.

Predicting the timing of events enhances the perceptual processing of those events. Jones et al. (2002) demonstrated that the discrimination of pitch was enhanced when a target sound was located at the last of regular rhythm so that listeners could predict its timing. Such rhythmic prediction may also be involved with improvements in temporal sensitivity, as found in the *multiple-look effect* (Schulze, 1989; Drake and Botte, 1993; Miller and McAuley, 2005; Ten Hoopen et al., 2011). This effect is typically tested with three successive sounds that delimit two neighboring empty durations, namely the standard (S) and the comparison duration (C). Discrimination of these durations is improved when the presentation of the standard duration is repeated before the comparison (as SSSSC; see Schulze, 1989; Ten Hoopen et al., 2011; Li et al., 2016).

The multiple-look effect is usually explained by a statistical model in which the variability of perceived duration of the standard reduces with an increase in the number of observations. In other words, if the perceived duration is averaged across observations (within a trial within an individual), the standard error of the mean is reduced as the number of observations is increased (Miller and McAuley, 2005; Li et al., 2016). These models then indicate that the size of the multiple-look effect is mainly determined by the number of observations as well as some weighting parameters. Therefore, there is no reason to expect a change in its size, whether the standard is repeated before or after the comparison (i.e., whether the sequence is SSSSC or CSSSS). This idea seems consistent with that reported by several studies in which the multiple-look effect also occurs when the standard is repeated after the comparison (Miller and McAuley, 2005; Ten Hoopen et al., 2011).

However, Ten Hoopen et al. (2011) provided a compelling evidence, in their third experiment, that the multiple-look effect is stronger when the standard is repeated before the comparison than when the standard is repeated after the comparison^1^. This difference is difficult to explain with the statistical model. Furthermore, in Drake and Botte (1993), the multiple-look effect was reduced when the regularity of sounds in the consecutive standards, as well as in the consecutive comparisons, was broken. Given this finding, we assume not only the statistical mechanism but also the prediction based on regular rhythm underlying the multiple-look effect. In the latter mechanism, listeners perceive regular rhythm during the repetition of the standard, predict the timing of subsequent sounds, and detect a sound that is displaced from the predicted timing, thus improving temporal sensitivity.

This prediction-based explanation falls into the frameworks of the *dynamic attending theory* (Jones and Boltz, 1989; Jones et al., 2002; McAuley and Jones, 2003; McAuley and Fromboluti, 2014). This theory indicates that the attentional level is not static but dynamically changes in time. The most recent version of the theory posits that the attentional level periodically oscillates, and that this attentional oscillation is entrained by the rhythm of external stimuli (Jones et al., 2002; McAuley and Jones, 2003; McAuley and Fromboluti, 2014). In other words, the attentional level synchronizes its peak with successive stimuli if those stimuli appear at regular intervals. For stimulus sequences causing the multiple-look effect (as SSSSC), the attentional oscillation is entrained during the repetition of the standard. This entrained oscillation enables listeners to discriminate the standard and the comparison based on whether the last stimulus of the comparison appears earlier than, later than, or simultaneously with the peak of the oscillation. Such benefits are not given when the standard is repeated after the comparison (as CSSSS).

**Figure 1:**
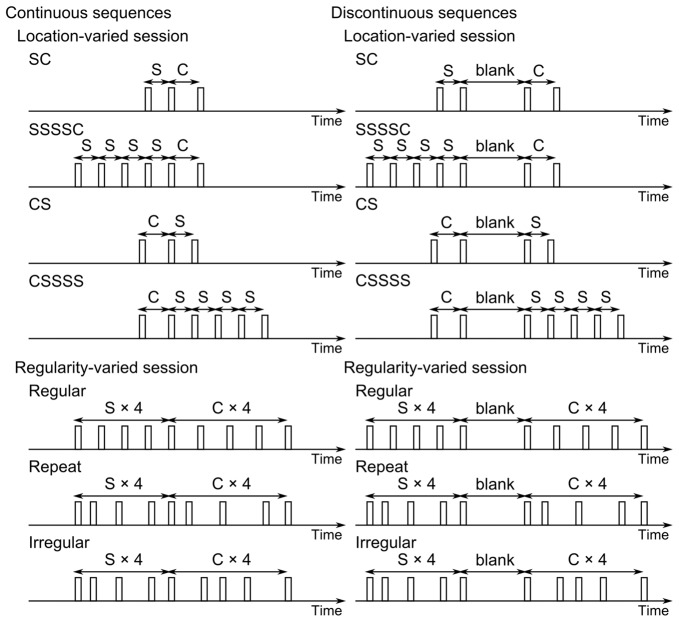
Stimuli used in the present study. The standard duration (S) was 200, 300 or 400 ms. The comparison duration (C) was 60 ms (140 ms in training) briefer or longer than S. The blank duration was 3.0 to 3.5 times of S.

The present experiment was conducted to examine whether the multiple-look effect would occur when the standard and the comparison were separated by a temporal blank (as SSSS_C; see Figure 1). If the attentional entrainment contributes to the occurrence of the multiple-look effect, a temporal blank between the standard and the comparison should violate the listener’s prediction based on regular rhythm because sounds are lacking at the predicted timing, thus resetting the attentional oscillation. In this article, the sequences in which the standard and the comparison are adjacent (as SSSSC) are called the “continuous” sequences while the sequences in which the standard and the comparison are separated (as SSSS_C) are called the “discontinuous” sequences.

There are two hypotheses: (1) if the multiple-look effect is only attributed to the statistical reduction of variability with an increase in the number of the standard, it should take place, whether or not the standard and the comparison are temporally separated, resulting in no differences between the continuous and discontinuous sequences. (2) If the multiple-look effect is also sourced from the rhythmic prediction, a temporal blank should violate the listener’s prediction based on regular rhythm, resulting in a lower performance in the discontinuous than the continuous sequences. However, this blank effect should be observed only when the standard is repeated before the comparison (SSSSC vs. SSSS_C). In other words, it should not be observed when the standard is repeated after the comparison (CSSSS vs. C_SSSS) because this condition does not benefit from the rhythmic prediction. Similarly, no blank effect should be observed when the regularity of sounds in the consecutive standards is broken, as depicted in Figure 1 (in “repeat” and “irregular”).

No studies have yet compared the continuous vs. discontinuous sequences to test the occurrence of the multiple-look effect, except Grondin (2001) using visual stimuli. This author reported that the multiple-look effect occurred with the discontinuous sequences but not with the continuous ones. In the auditory modality, the continuous sequences (Schulze, 1989; Ten Hoopen et al., 2011; Li et al., 2016) and the discontinuous ones (Drake and Botte, 1993; Miller and McAuley, 2005; Grondin, 2012) have been used in separate studies. The present study is the first one comparing the continuous vs. discontinuous sequences directly with auditory stimuli.

Drake and Botte (1993) and Miller and McAuley (2005) demonstrated the occurrence of the multiple-look effect with discontinuous sequences in the auditory modality, and this result does not seem to support our prediction-based hypothesis. However, in their experiments, the standard and the comparison were separated by a blank that was twice as long as the standard duration. In this case, only one sound was missing at the predicted timing between the last sound of the standard and the first of the comparison. The blank thus might have been too short to reset the attentional oscillation. The oscillation then continued after the blank, and its peak appeared coincidently with the first stimulus of the comparison, keeping the multiple-look effect. In the present experiment, a longer blank was adopted to enhance the effects of resetting the attentional oscillation. The standard and the comparison were separated by an inter-onset interval lasting three times or more of the standard; thus, two sounds were lacking at the predicted timing between the last sound of the standard and the first of the comparison. Furthermore, the duration of the blank was jittered across trials to prevent listeners from memorizing the duration and thus compensating the blank in their mind such that the attentional oscillation could be kept (see McAuley and Kidd, 1998).

**Figure 2:**
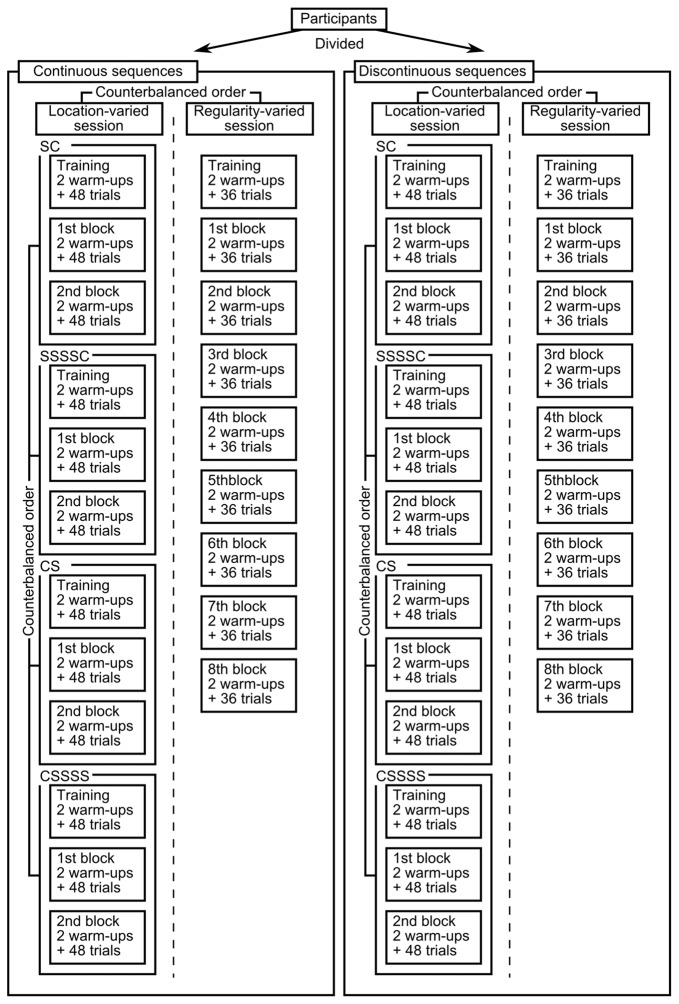
Experimental structures.

The present experiment consisted of two sessions (Figure 2). In the location-varied session, the position of the standard and comparison (SC vs. CS) and the number of presentation of the standard (1 vs. 4) were manipulated, as in Miller and McAuley (2005) and Ten Hoopen et al. (2011). In the regularity-varied session, the standard and the comparison were presented four times each, and the regularity of sounds in the consecutive standards, as well as in the consecutive comparisons, was manipulated, as in Drake and Botte (1993). As a novel parameter, the sequence continuity (continuous vs. discontinuous) was examined in both sessions, based on the hypotheses mentioned above.

## Materials and Methods

### Participants, Ethics and Experimental Structures

The experiment was conducted in accordance with Declaration of Helsinki as well as with procedures approved by the ethics review board of Shizuoka University. Written informed consent was obtained from each participant.

Sixteen participants that self-reported having normal hearing were recruited and divided into two groups. Eight of them (1 female), aged 20–23 years, listened to the continuous sequences, while the others (2 females), aged 19–23 years, listened to the discontinuous sequences. Thus, the continuity effect was tested with a between-participants design. The other effects were tested with a within-participants design.

Each group performed both the location-varied and regularity-varied sessions, as depicted in Figure 2. Half (4) participants of each group performed first the location-varied session and then the regularity-varied session, whereas the others performed these sessions in the opposite order. Participants assigned to the continuous sequences took about 2 h to complete the experiment, and those assigned to the discontinuous sequences took about 2.5 h.

### Location-Varied Session

#### Stimuli and Apparatus

The location-varied session consisted of three independent variables: location, continuity and standard duration. The first two were of interest to examine the statistical and prediction-based hypotheses. The first variable represents whether the standard was repeated before or after the comparison (There were also the control conditions in which the standard was presented only once). The second represents whether the standard and the comparison were adjacent to each other or separated by a temporal blank. If the multiple-look effect is just a matter of the statistical reduction of variability with an increase in the number of the standard, it should occur, whether or not the standard and the comparison are separated. However, if the rhythmic prediction contributes to the multiple-look effect, inserting a blank between the standard and the comparison should violate the listener’s prediction based on regular rhythm and reduce the multiple-look effect. However, this reduction should be found only when the standard is repeated before the comparison but not when it is repeated after the comparison because the latter condition does not benefit from the rhythmic prediction. Finally, the last variable represents how long the standard was, and this manipulation was adopted to check if the continuity and location effects would be found in a specific duration or not.

There were four location conditions. In SC, a standard interval (S) preceded a comparison interval (C). In SSSSC, four standard intervals were successively presented before a comparison interval. In CS, a comparison interval preceded a standard interval. In CSSSS, a comparison interval was presented before four standard intervals.

The standard duration was varied in three conditions: S = 200, 300 or 400 ms. As explained later, participants were asked to compare the standard and the comparison, and there was a 60-ms difference between these intervals: C = S + 60 or S − 60 ms. The duration was manipulated in terms of an inter-onset interval (i.e., an interval between the onsets of two successive sounds).

There were two continuity conditions. In the continuous condition, the standard and the comparison were adjacent to each other. In the discontinuous condition, they were separated by a temporal blank. For example, the continuous SC consisted of three sounds; the first and second sounds delimited the standard, and the second and third ones delimited the comparison. However, the discontinuous SC consisted of four sounds; the first and second sounds delimited the standard, and the third and fourth sounds delimited the comparison. For the latter case, a blank between the second and third sounds lasted *k* × S (200, 300 or 400 ms). *k* was randomly varied between 3 and 3.5 across trials, as mentioned in “Introduction” section, to prevent listeners from memorizing the duration and thus compensating the blank in their mind such that the attentional oscillation could be kept (McAuley and Kidd, 1998).

Each sound was a sinusoid of 1000 Hz and 70 dB SPL. Its length was 10 ms, including the rise and the decay ramps of 4 ms with raised-cosine windows to avoid spectral splatter. Digital signals of stimuli were sampled at 44,100 Hz and quantized to 16 bits. These signals were converted into analog ones by a USB DAC (Onkyo SE-U33GXVII) and were presented from headphones (Sennheiser HD 380 pro) connected to an amplifier (Teac A-H01).

#### Procedure

The four location conditions (SC, SSSSC, CS and CSSSS) were presented in separate sub-sessions, resulting in four sub-sessions in the location-varied session (Figure 2). The order of these sub-sessions was counterbalanced: the possible order was SC-SSSSC-CS-CSSSS, SSSSC-SC-CSSSS-CS, CS-CSSSS-SC-SSSSC, or CSSSS-CS-SSSSC-SC.

Before each sub-session, participants were given a schematic explanation of stimulus sequences (as in Figure 1) as well as the definition of two intervals to be discriminated. We did not use the terms “standard” and “comparison” when instructing the task to participants. We instead asked them to judge whether “d2” was shorter or longer than “d1”. For the SC and SSSSC sequences, d1 and d2 indicated the standard and the comparison, respectively, but for the CS and CSSSS sequences, d1 and d2 indicated the comparison and the standard, respectively. Thus, d1 was always followed by d2, making the task of participants consistent throughout the session (thus preventing confusions). Participants pushed the left button of a computer mouse to respond that d2 was “shorter” than d1 and the right button to respond that d2 was “longer” than d1. The next trial began 1–2 s after the response.

Each sub-session included two blocks. In each block, six stimulus sequences (= 3 standards × 2 comparisons) were presented eight times each, resulting in 48 trials. The order of trials was randomized with a restriction that an identical sequence was not presented in two consecutive trials. A few-seconds break was taken between the blocks. Two warm-up trials in which randomly selected conditions were presented were conducted before the beginning of each block.

A training block was conducted before the beginning of each sub-session. This training included two warm-ups plus 48 trials as in the experimental block. However, the duration difference between the standard and the comparison was 140 ms instead of 60 ms, and the participant’s response was followed by a feedback message indicating whether the response was correct or incorrect. For example, “correct” was presented on a computer display when participants responded “longer” for sequences in which d2 was physically longer than d1, whereas “incorrect” was presented when participants responded “shorter” for sequences in which d2 was physically longer than d1.

### Regularity-Varied Session

#### Stimuli and Apparatus

The regularity-varied session consisted of three independent variables; regularity, continuity and standard duration. The last two were also adopted in the location-varied session, but a sharper focus was put on the first variable in this session. As shown in Figure 1, each sequence consisted of the four-standards pattern and the four-comparisons pattern. The regularity of sounds included in each pattern was manipulated. The prediction-based hypothesis expects the occurrence of the multiple-look effect if sounds are aligned at regular intervals and if no blank is inserted between the standard and the comparison pattern. If the regularity of sounds in each pattern is broken (i.e., if those sounds are aligned at irregular intervals in each pattern), the rhythmic prediction should not work, resulting in no multiple-look effect, in the continuous as well as the discontinuous sequences.

There were three regularity conditions (Figure 1). In the *regular* condition, sounds were aligned at regular intervals in each pattern. In the *repeat* and *irregular* conditions, sounds were aligned at irregular intervals in each pattern. However, in the repeat condition, the ratios of intervals in the standard pattern were identical to those in the comparison pattern. Therefore, this condition had a repetition of identical (interval-ratio) structures, resulting in regularity in a higher level of rhythmic hierarchy (Jones and Boltz, 1989), and might benefit from the rhythmic prediction even though to a lesser extent than the regular condition.

The following are the technical details of the regularity manipulation. In the regular condition, the standard pattern consisted of four identical intervals, each one (S) being 200, 300 or 400 ms. The comparison pattern consisted of four identical intervals, each one (C) being 60 ms shorter or longer than S.

In the repeat condition, the standard pattern consisted of an interval lasting S × 0.6, an interval lasting S × 0.9, an interval lasting S × 1.1, and an interval lasting S × 1.4. The possible order of these intervals was listed in Table 1; an order was randomly chosen across trials. The comparison pattern had the same order as the standard pattern, except that S was replaced by C (= S + 60 or S − 60 ms); thus, both patterns had the same interval-ratio structure. Note that the list did not include accelerating and decelerating structures, such as [×1.4]−[×1.1]−[×0.9]−[×0.6] and [×0.6]−[×0.9]−[×1.1]−[×1.4], as well as structures in which shorter and longer intervals were alternated, e.g., [×0.6]−[×1.1]−[×0.9]−[×1.4], because they might have yielded a specific impression of regularity.

**Table 1:** Intervals of the standard and comparison patterns for the repeat and irregular conditions.

	Repeat							
	Standard pattern				Comparison pattern			
No.	1st	2nd	3rd	4th	1st	2nd	3rd	4th
1	0.6	1.1	1.4	0.9	0.6	1.1	1.4	0.9
2	0.6	1.4	1.1	0.9	0.6	1.4	1.1	0.9
3	0.9	1.1	1.4	0.6	0.9	1.1	1.4	0.6
4	0.9	1.4	1.1	0.6	0.9	1.4	1.1	0.6
5	1.1	0.6	0.9	1.4	1.1	0.6	0.9	1.4
6	1.1	0.9	0.6	1.4	1.1	0.9	0.6	1.4
7	1.4	0.6	0.9	1.1	1.4	0.6	0.9	1.1
8	1.4	0.9	0.6	1.1	1.4	0.9	0.6	1.1
	**Irregular**							
	**Standard pattern**				**Comparison pattern**			
**No.**	**1st**	**2nd**	**3rd**	**4th**	**1st**	**2nd**	**3rd**	**4th**
1	0.6	1.1	1.4	0.9	1.1	0.6	0.9	1.4
2	0.6	1.4	1.1	0.9	1.4	0.6	0.9	1.1
3	0.9	1.1	1.4	0.6	1.1	0.9	0.6	1.4
4	0.9	1.4	1.1	0.6	1.4	0.9	0.6	1.1
5	1.1	0.6	0.9	1.4	0.6	1.1	1.4	0.9
6	1.1	0.9	0.6	1.4	0.9	1.1	1.4	0.6
7	1.4	0.6	0.9	1.1	0.6	1.4	1.1	0.9
8	1.4	0.9	0.6	1.1	0.9	1.4	1.1	0.6

*Note. The standard duration (S) and comparison duration (C) were multiplied by the coefficient indicated in this table. For example, no. 1 means that the first interval of the standard lasted S × 0.6, the second lasted S × 1.1, the third lasted S × 1.4, and the last lasted S × 0.9*.

In the irregular condition, the same method as in the repeat condition to manipulate each interval was adopted. However, as indicated in Table 1, the standard and comparison patterns had different structures; the first and second intervals in the standard pattern were interchanged with each other in the comparison pattern, and so were the third and fourth intervals.

The standard and comparison patterns were adjacent to each other in the continuous condition while they were temporally separated in the discontinuous condition. For the latter case, the last sound of the standard and the first sound of the comparison was separated by *k* × S (200, 300 or 400 ms). *k* was randomly varied between 3 and 3.5 across trials. The same apparatus and sounds as in the location-varied session were used.

#### Procedure

Participants were instructed to judge whether the last half of sounds (in the comparison pattern) were presented in a “faster” or “slower” tempo than the first half (in the standard pattern) in each trial. The session consisted of eight blocks. In each block, 18 stimulus patterns (= 3 regularities × 3 standards × 2 comparisons) were presented twice, resulting in 36 trials. The order of trials was randomized with a restriction that an identical sequence was not presented in two consecutive trials. A few-seconds break was taken between the blocks. Two warm-up trials in which randomly selected sequences were presented were conducted before the beginning of each block.

A training block was conducted before the first experimental block. This training included two warm-ups plus 36 trials as in the experimental block. Furthermore, the difference between S and C was 140 ms instead of 60 ms, and the participant’s response was followed by a feedback message.

### Data Analysis and Statistics

The data of the location-varied session and the regularity-varied session were analyzed with the same methods. The warm-up trials and the training blocks were removed from the analysis.

*d′* was estimated based on the signal detection theory (Stanislaw and Todorov, 1999; MacMillan and Creelman, 2005) to examine temporal sensitivity, following previous studies in time perception (Schulze, 1989; Grondin, 1998; Kuroda and Grondin, 2013; Kuroda et al., 2016)^2^. This dependent variable expresses how well participants discriminated between the −60 ms and the +60 ms comparison interval; a higher value indicates better discrimination. It was calculated with the following equation:

(1) $$d^{\prime }\;=\;\Phi^{-1}(H)\;-\;\Phi^{-1}(F)$$

Φ^−1^ (*H*) is a *z* score of the hit probability and Φ^−1^ (*F*) is a *z* score of the false-alarm probability.

In the location-varied session, the hit probability means how frequently participants responded “longer” when the comparison was physically longer than the standard. The false-alarm probability means how frequently participants responded “longer” when the comparison was physically shorter than the standard. Note that, since participants were asked to judge whether d2 was shorter or longer than d1, they responded “longer” for the SC and SSSSC conditions when the comparison (d2) was perceived as longer than the standard (d1), but responded “shorter” for the CS and CSSSS conditions when the comparison (d1) was perceived longer than the standard (d2). We, therefore, read “shorter” for the CS and CSSSS condition as meaning “longer” in the data analysis.

In the regularity-varied session, the hit probability means how frequently participants responded “slower” when the comparison was physically longer than the standard. The false-alarm probability means how frequently participants responded “slower” when the comparison was physically shorter than the standard.

For both sessions, each probability was based on 16 responses for each condition for each participant. Furthermore, the *log-linear* method was adopted to correct each probability to avoid obtaining extreme values (0 and 1) which led to infinite when converted into the *z* score (Hautus, 1995).

The location-varied session was based on a 2 (continuities) × 4 (locations) × 3 (standards) design with repeated measures for the last two factors. The regularity-varied session was based on a 2 (continuities) × 3 (regularities) × 3 (standards) design with repeated measures for the last two factors. An analysis of variance (ANOVA) was conducted for each session. *F* distribution was estimated with the degrees of freedom that were corrected by the Greenhouse-Geisser epsilon against potential violation of sphericity. When the interaction was significant, the simple main effect was tested by a one-way ANOVA with the Greenhouse-Geisser correction. Pairwise contrasts were conducted based on the Holm method when the main or the simple main effect was significant.

## Results

### Location-Varied Session

**Figure 3:**
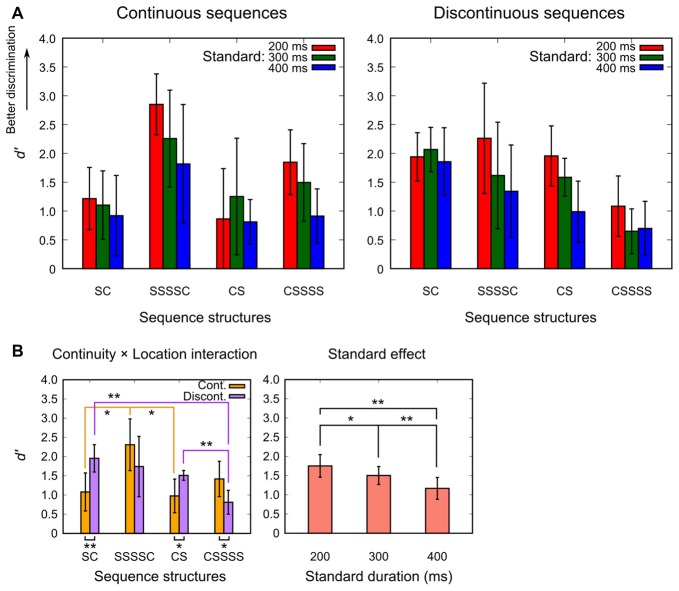
Mean *d′* for each experimental condition of the location-varied session **(A)**, and the results of the *post hoc* contrasts for the continuity × location interaction and the standard effect that were significant in the omnibus analysis of variance (ANOVA) **(B)**. Bars represent 95% confidence intervals. Asterisks indicate significant differences (*p < 0.05, **p < 0.01).

The mean *d′* for each experimental condition in the location-varied session is shown in Figure 3. In general, the SSSSC conditions yielded the highest *d′* (sensitivity) in the continuous sequences, whereas the CSSSS conditions yielded the lowest *d′* in the discontinuous sequences. Indeed, the ANOVA revealed that the location effect, *F*_(1.63,22.85)_ = 11.115, *p* < 0.001, $\eta_{\text{p}}^{2}$ = 0.443, as well as its interaction with the continuity effect, *F*_(1.63,22.85)_ = 9.866, *p* = 0.001, $\eta_{\text{p}}^{2}$ = 0.413, was significant. The standard effect was also significant, *F*_(1.62,22.67)_ = 15.365, *p* < 0.001, $\eta_{\text{p}}^{2}$ = 0.523. No other effects were significant (*p* > 0.259).

The results of the *post hoc* contrasts, as shown in Figure 3, are summarized as follows: (1) the SSSSC condition resulted in a higher *d′* than the control (SC and CS) conditions, indicating the occurrence of the multiple look effect, but only in the continuous sequences. (2) The CSSSS condition resulted in a lower *d′* than the control (SC and CS) conditions in the discontinuous sequences. (3) The 200-ms standard yielded the highest *d′* and was followed by the 300-ms and then the 400-ms standard.

### Regularity-Varied Session

**Figure 4:**
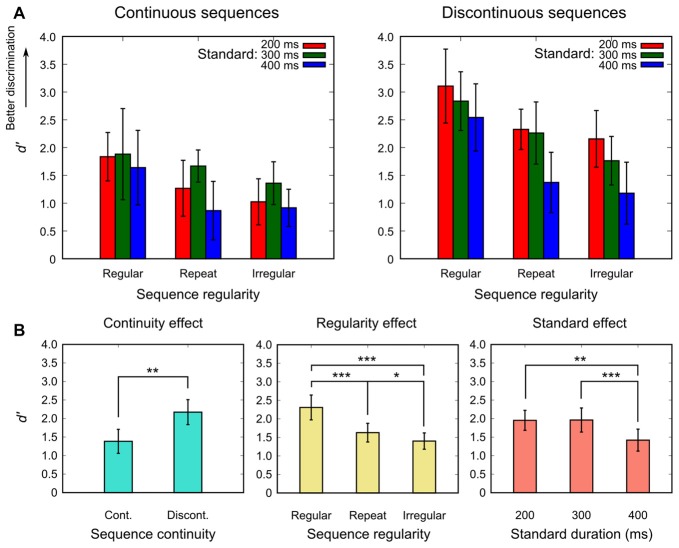
Mean *d′* for each experimental condition of the regularity-varied session **(A)**, and the results of the *post hoc* contrasts for all main effects that were significant in the omnibus ANOVA **(B)**. Bars represent 95% confidence intervals. Asterisks indicate significant differences (*p < 0.05, **p < 0.01, ***p < 0.001).

The mean *d′* for each experimental condition in the regularity-varied session is shown in Figure 4. In general, *d′* was the highest for the regular condition. The ANOVA revealed that all main effects were significant: the continuity effect, *F*_(1,14)_ = 15.891, *p* = 0.001, $\eta_{\text{p}}^{2}$ = 0.532, the regularity effect, *F*_(1.69,23.72)_ = 33.824, *p* < 0.001, $\eta_{\text{p}}^{2}$ = 0.707, and the standard effect, *F*_(1.86,26.03)_ = 12.759, *p* < 0.001, $\eta_{\text{p}}^{2}$ = 0.477. No interactions were significant (*p* > 0.051).

The results of the *post hoc* contrasts, as shown in Figure 4, are summarized as follows: (1) the regular condition resulted in the highest *d′* and was followed by the repeat and then the irregular condition. (2) *d′* was higher for the discontinuous than for the continuous sequences. (3) The 200-ms as well as the 300-ms standard yielded a higher *d′* than the 400-ms standard.

## Discussion

### Statistical vs. Prediction-Based Mechanisms (in the Location-Varied Session)

The present study was conducted to examine whether the multiple-look effect would occur when the standard and the comparison were separated by a temporal blank, based on two hypotheses. The first hypothesis attributes the multiple-look effect to the statistical reduction of variability with an increase in the number of the standard, and this does not expect any differences between the continuous vs. discontinuous sequences. The second hypothesis attributes the multiple-look effect to the listener’s prediction based on regular rhythm, and this expects that a temporal blank between the standard and the comparison resets the attentional oscillation, thus inhibiting the multiple-look effect.

The results of the location-varied session may be evidence against the statistical hypothesis. For the continuous sequences, the SSSSC condition yielded a higher performance than the control (SC and CS) conditions, indicating the occurrence of the multiple-look effect. However, for the discontinuous sequences, the SSSSC and control conditions led to almost identical performances. If the multiple-look effect had been just a matter of the statistical reduction of variability, this effect should have occurred, whether or not the standard and the comparison were separated by a temporal blank.

The results instead seemed consistent with those expected from the prediction-based hypothesis. For the SSSSC condition, the attentional oscillation was entrained during the repetition of the standard. When the comparison was adjacent to the preceding standard (i.e., in the continuous sequences), participants could utilize this entrained oscillation for discrimination; they discriminated between the standard and the comparison based on whether the last sound of the comparison appeared earlier or later than the peak of the oscillation. However, when a temporal blank was inserted between the standard and the comparison (i.e., in the discontinuous sequences), the attentional oscillation was rest during the blank, canceling out the benefits from the attentional oscillation. Therefore, the multiple-look effect occurred in the continuous sequences but not in the discontinuous sequences.

### The Inversed Multiple-Look Effect (in the Location-Varied Session)

However, the CSSSS condition yielded a lower performance than the control (SC and CS) conditions when the comparison was separated from the subsequent standard (i.e., in the discontinuous sequences). The prediction-based hypothesis did not expect any effects in the CSSSS condition, in which the attentional oscillation could not help discrimination because the comparison was presented before the oscillation was entrained by the repetition of the standard. Therefore, both the continuous and discontinuous sequences should have yielded no multiple-look effect, i.e., identical performances, in the CSSSS condition. However, this condition indeed exhibited an impairment effect (lower sensitivity than SC and CS) for the discontinuous sequences, suggesting the occurrence of an *inversed* multiple-look effect.

One might explain this impairment effect by the decay of memory. Since participants responded after the presentation of the last stimulus, they had to sustain the memory of the comparison longer when a temporal blank was inserted between the comparison and the subsequent standard. Then, the memory was more likely decayed, impairing the discrimination performance. However, this explanation seems implausible because, in the control (SC and CS) conditions, the discontinuous sequences yielded a higher *d′* than the continuous sequences (Figure 3). For these conditions, the discontinuous sequences should have led to lower performances than the continuous sequences if the temporal blank of the present experiment had been long enough to facilitate the decay of memory.

Although speculative, the inversed multiple-look effect might be explained by adding two further assumptions to the prediction-based hypothesis: (1) the repetition of the standard after the comparison could be interference with the listener’s decision process before the response. For the C_SSSS (discontinuous) condition, listeners could not utilize the rhythmic prediction and thus simply compared the comparison and the first standard. This seemed to be the simplest strategy, but since the standard was consecutively repeated, listeners had to segregate the first standard from the others in their mind. This cognitive demand reduced the performance in the C_SSSS compared with the C_S condition. However, only with this assumption, it is difficult to explain why there were no differences between the CSSSS and CS (continuous) conditions. In the CSSSS condition, the repetition of the standard could have been interference. We therefore needed the next assumption. (2) Even during the presentation of only two sounds, the attentional oscillation could be entrained slightly and thus could be utilized for discrimination. In the CSSSS condition, the attentional oscillation was entrained during the presentation of the comparison, and even though this entrainment was weak, participants could discriminate the comparison and the standard based on whether the second sound of the first standard appeared earlier or later than the peak of the oscillation. The same strategy could be applied to the CS condition, resulting in an identical performance to the CSSSS condition.

In brief, the formation of regular rhythm produces two opposite effects; it both improves and impairs temporal sensitivity. The impairment effect seemed difficult to explain with the statistical hypothesis, but also required the prediction-based hypothesis to be revised much. A simpler, more comprehensive model should be constructed to explain the multiple-look effect and the inversed one.

### Border Effects (in the Regularity-Varied Session)

Inserting a blank between the standard and the comparison led to another effect in the regularity-varied session; the discontinuous sequences resulted in a higher *d′* than the continuous sequences (Figure 4). This result was unexpected from the statistical hypothesis as well as the prediction-based one. However, it is not surprising, given that in this session participants performed discrimination based on not only tempo but also the whole duration (i.e., between the beginning sound and the last sound) of the standard and the comparison pattern. For the discontinuous sequences, the borders (beginning and end) of each pattern were clear because the standard pattern and the comparison pattern were separated by a temporal blank. However, for the continuous sequences, the borders of each pattern were less clear because the end of the standard pattern and the beginning of the comparison pattern were delimited by an identical sound. Therefore, the whole duration of the standard pattern and that of the comparison pattern might be discriminated in the discontinuous sequences easier than in the continuous sequences.

Notably, this explanation could be applied to the results of the control (SC and CS) conditions in the location-varied session. These conditions also exhibited a higher *d′* for the discontinuous sequences than the continuous ones. The standard and comparison intervals were each delimited clearly when they were separated by a temporal blank (for the discontinuous sequences) but not when the end of the standard and the beginning of the comparison shared an identical sound (for the continuous sequences).

Nevertheless, there might be another approach explaining the results of the control conditions in the location-varied session. A temporal assimilation might occur in the continuous sequences; the perceived duration of the standard and the comparison were assimilated when those intervals were neighboring to each other (Nakajima et al., 2004; Grondin et al., 2017). This might result in a lower sensitivity for the continuous than the discontinuous sequences. However, such an assimilation typically occurs when the first interval is 200 ms or briefer (Nakajima et al., 2004), or when the total of the two intervals is 540 ms or briefer (Miyauchi and Nakajima, 2007). Only the 200-ms standard in the present experiment fulfilled these duration-range criteria.

### Prediction Based on Rhythmic Hierarchy (in the Regularity-Varied Session)

More important, in the regularity-varied session, the discrimination performance changed as a function of regularity; the regular condition yielded the highest sensitivity, and was followed by the repeat and then the irregular condition. Sounds were aligned irregularly in the last two conditions, but the standard and comparison patterns had the same interval-ratio structure in the repeat condition. In other words, the repeat condition had a repetition of identical structures (i.e., regularity) in a higher hierarchical level. The result thus suggests that temporal sensitivity benefits from the formation of rhythm in any hierarchical level whereas the benefits become smaller with a more complex structure of hierarchy (Jones and Boltz, 1989).

### Relation with the Oddball Paradigm and Potential Integration of Two Mechanisms

We have contrasted the statistical vs. prediction-based hypotheses, but it might be possible to assume these hypotheses as complementary to each other. The results of the location-varied session obviously indicate that the temporal relationships between the standard and the comparison are a crucial factor determining the multiple-look effect. The statistical hypothesis has not taken this factor into account. However, it is also true to say that there are several researches demonstrating a good fitting of the statistical model to behavioral data (Schulze, 1989; Miller and McAuley, 2005; Ten Hoopen et al., 2011; Li et al., 2016) even when the standard was repeated after the comparison (Miller and McAuley, 2005; Ten Hoopen et al., 2011). Given this, it would be reasonable to posit that the statistical mechanism can also work in the multiple-look effect, but the prediction-based mechanism is dominant in some cases (as in the present experiment), resulting in a stronger effect when the standard is repeated before the comparison than when repeated after the comparison.

In order to discuss the potential integration of the two hypotheses, it seemed worth noting that the stimulus sequences used in the present experiment are very similar to those used in neurophysiological studies with the oddball paradigm (for review, see Garrido et al., 2009). An oddball stimulus that is deviated, for example, in pitch or duration from the other sequential stimuli elicits the mismatch negativity (MMN) that is recorded with electroencephalography (EEG) or magnetoencephalography (MEG). Psychophysical studies have also shown that the perceived (filled) duration of the oddball stimulus is distorted (typically overestimated) compared with that of the non-oddball ones (Tse et al., 2004; Pariyadath and Eagleman, 2007; McAuley and Fromboluti, 2014). In the present experiment, the last sound of the comparison in the SSSSC condition can be regarded as the oddball. This sound might have activated neural responses reflected by MMN. Indeed, MMN is interpreted to be evoked when the regularity of successive sounds is broken and generated by a neural process comparing the current sensory input with a memory trace of previous stimuli (Garrido et al., 2009). If the activation level of those neural responses increases as a function of the number of the standard and correlates with temporal sensitivity, it determines the occurrence of the multiple-look effect. This explanation is compatible with both the statistical and the prediction-based hypothesis. Therefore, further investigations of the multiple-look effect with neurophysiological techniques will give new insights into understanding the duration processing of successive intervals.

## Conclusion

We have discussed the results of the present experiment, focusing on the validity of the statistical vs. prediction-based hypotheses that explain the multiple-look effect. Inserting a temporal blank between the standard and the comparison produced several effects that the statistical hypothesis did not expect. The multiple-look effect resulting from the repetition of the standard before the comparison was diminished when the standard and the comparison were separated by a blank, supporting the prediction-based hypothesis. However, this hypothesis required a lot of revisions to explain an impairment effect that was observed when the standard was repeated after the comparison. In this condition, discrimination was impaired when a temporal blank was inserted between the comparison and the subsequent standard. Further investigations with neurophysiological techniques recording MMN may give a comprehensive theory explaining the multiple-look effect and the inversed one. A recent neurophysiological study reported that the multimodal training, including motor production, of musical rhythm induces the cortical plasticity involved with the improvements of temporal sensitivity (Lappe et al., 2011). The current finding that the formation of regular rhythm both improves and impairs temporal sensitivity might be an addition to the literature in clinical fields to find an effective method utilizing rhythmic activities to adjust the human time performance.

## Author Contributions

TK, DY and MM conceived the project. TK designed and prepared the experiments. TK, TU and DY collected the data. TK and DY analyzed the data. TK drafted the article. All authors revised the article.

## Conflict of Interest Statement

The first author, TK, is currently employed by Yamaha Motor Co., Ltd. The study presented in this article was conducted when TK was employed by Shizuoka University (until March 2017). The other authors declare that the research was conducted in the absence of any commercial or financial relationships that could be construed as a potential conflict of interest.

## Figures and Tables

**Figure 5:**
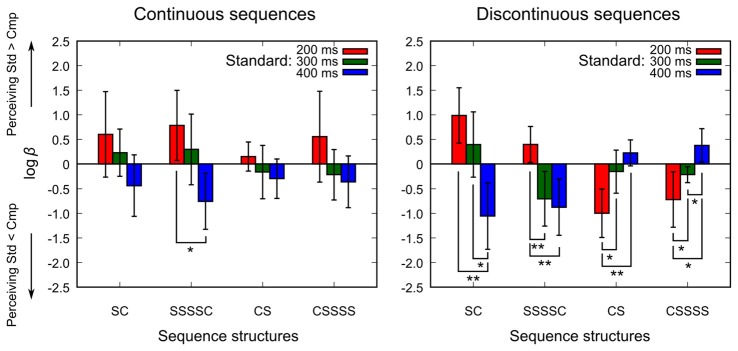
Mean log *β* for each experimental condition of the location-varied session. Since the three-way interaction was significant, the *post hoc* contrasts were conducted without pooling any factors. Bars represent 95% confidence intervals. Asterisks indicate significant differences (*p < 0.05, **p < 0.01).

**Figure 6:**
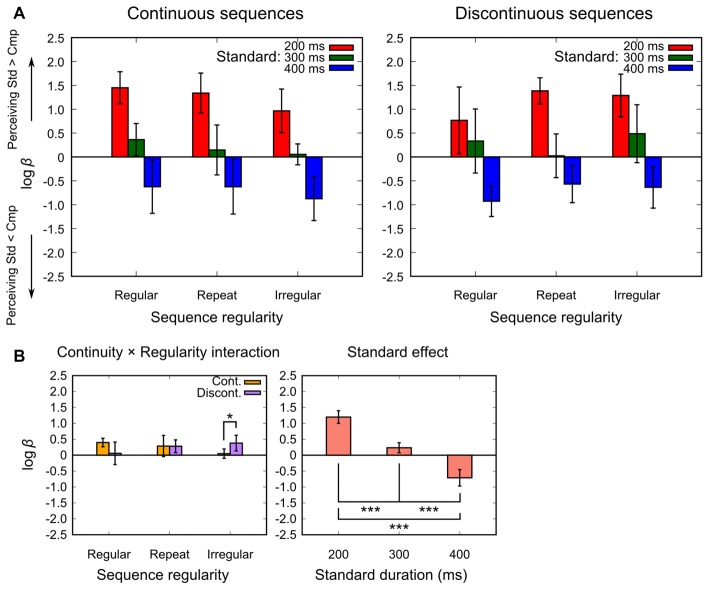
Mean log *β* for each experimental condition of the regularity-varied session **(A)**, and the results of the *post hoc* contrasts for the continuity × regularity interaction and the standard effect that were significant in the omnibus ANOVA **(B)**. Bars represent 95% confidence intervals. Asterisks indicate significant differences (*p < 0.05, ***p < 0.001).

## Footnotes

^1^More precisely, these authors manipulated the number of the standard and that of the comparison independently. The standard preceded (SC) or followed (CS) the comparison. The repetition of the standard for SC and of the comparison for CS led to a stronger multiple-look effect than that of the comparison for SC and of the standard for CS. Note that in the present article only the number of the standard was manipulated as an independent variable.

^2^See Appendix, (Figures 5, 6) for the results of *β* expressing perceived duration.

## Appendix: Perceived Duration

*β* was also estimated, expressing how long participants perceived the comparison duration, in comparison with the standard. A lower value indicates more “longer (slower)” responses than “shorter (faster)” ones. Note that *β* is usually used in detection tasks to express the tendency for participants to prefer responding one of the two alternatives. However, in the present study, this measure is interpreted as a sign of perceived duration; for example, if duration is perceived as longer, participants should respond “longer” more frequently than “shorter” (Grondin, 1998; Kuroda et al., 2016). *β* was calculated by the following equation:

(1) $$log\;\beta \;=\;\frac{{\left[\Phi^{-1}(F)\right]}^{2}\;-\;{\left[\Phi^{-1}(H)\right]}^{2}}{2}$$

The natural logarithm was adopted for keeping the linearity of scale. log *β* was zero when the number of “shorter (faster)” responses was equal to that of “longer (slower)” responses; i.e., the standard and the comparison were perceived as equivalent. log *β* was higher than zero when participants were likely to perceive the comparison duration (or tempo) as shorter (faster) than the standard, whereas it was lower than zero when they were likely to perceive the comparison as longer (slower) than the standard.

The mean log *β* for each experimental condition of the location-varied session is shown in Figure 5. An ANOVA according to a 2 (continuities) × 4 (locations) × 3 (standards) with repeated measures for the last two factors revealed that the standard effect as well as the interactions involved with this factor were significant: the standard effect, *F*_(1.95,27.35)_ = 13.574, *p* < 0.001, $\eta_{\text{p}}^{2}$ = 0.492, the continuity × standard interaction, *F*_(1.95,27.35)_ = 4.860, *p* = 0.016, $\eta_{\text{p}}^{2}$ = 0.258, the location × standard interaction, *F*_(3.92,54.84)_ = 9.011, *p* < 0.001, $\eta_{\text{p}}^{2}$ = 0.392, and the three-way interaction, *F*_(3.92,54.84)_ = 4.875, *p* = 0.002, $\eta_{\text{p}}^{2}$ = 0.258. No other effects were significant (*p* > 0.056).

Since the three-way interaction was significant, the *post hoc* contrasts were conducted without pooling any factors, as shown in Figure 5. Note that this figure indicates only the results of the contrasts among the standard conditions; the results of the other contrasts delivered almost the same messages. The results are summarized as follows: (1) for the discontinuous sequences, a longer standard yielded a lower log *β* in the SC and SSSSC conditions, but yielded a higher log *β* in the CS and CSSSS conditions. (2) For the continuous sequences, only the SSSSC condition exhibited the standard effect in which log *β* was decreased as the standard was lengthened.

The mean log *β* for each experimental condition of the regularity-varied session is shown in Figure 6. An ANOVA according to a 2 (continuities) × 3 (regularities) × 3 (standards) with repeated measures for the last two factors revealed that the standard effect was significant, *F*_(1.82,25.46)_ = 71.291, *p* < 0.001, $\eta_{\text{p}}^{2}$ = 0.836, and the continuity × regularity interaction was significant, *F*_(1.97,27.63)_ = 4.842, *p* = 0.016, $\eta_{\text{p}}^{2}$ = 0.257. No other effects were significant (*p* > 0.189).

The results of the *post hoc* contrasts, as shown in Figure 6, are summarized as follows: (1) log *β* was decreased as the standard was lengthened. (2) The discontinuous sequences produced a higher log *β* than the continuous sequences in the irregular condition.

In summary, for both sessions, the standard duration was the main factor determining the perceived duration of the comparison relative to the standard interval. There seemed to be no clear relationship between temporal sensitivity (*d′*) and perceived duration (log *β*) in the present study.
